# Nanotransfersomes-loaded thermosensitive *in situ* gel as a rectal delivery system of tizanidine HCl: preparation, *in vitro* and *in vivo* performance

**DOI:** 10.1080/10717544.2016.1245369

**Published:** 2017-02-03

**Authors:** Fatma A. Moawad, Adel A. Ali, Heba F. Salem

**Affiliations:** Department of Pharmaceutics, Faculty of Pharmacy, Beni-Suef University, Beni-Suef, Egypt

**Keywords:** Tizanidine HCl, transfersomes, rectal, *in situ* gelling systems, pharmacokinetics

## Abstract

The purpose of the current study was to develop tizanidine HCl (TIZ; a myotonolytic agent used for treatment of spasticity) loaded nanotransfersomes intended for rectal administration, aiming to bypass the hepatic first-pass metabolism. TIZ-loaded nanotransfersomes were prepared by thin-film hydration method followed by characterization for various parameters including entrapment efficiency, vesicle diameter, *in vitro* release and *ex vivo* permeation studies. Transfersomal formulation composed of phosphatidylcholine and Tween 80 at a weight ratio of (85:15) gave a satisfactory results. It exhibited encapsulation efficiency of 52.39%, mean diameter of 150.33 nm, controlled drug release over 8 h and good permeation characteristics. Optimum formula was then incorporated into Pluronic-based thermoreversible gel using hydroxypropyl methylcellulose (HPMC) as a mucoadhesive polymer. Pharmacokinetic study was performed by rectal administration of transfersomes-loaded *in situ* gel to rabbits and compared with oral drug solution and rectal TIZ *in situ* gel. The pharmacokinetic study revealed that the transfersomal formulation successively enhanced the bioavailability of TIZ by about 2.18-fold and increased *t*_1/2_ to about 10 h as compared to oral solution. It can be concluded that encapsulation of TIZ into nanotransfersomes can achieve a dual purpose of prolonged TIZ release and enhanced bioavailability and so may be considered as a promising drug delivery system for the treatment of spasticity.

## Introduction

Tizanidine HCl (TIZ) is a myotonolytic agent used for treatment of spasticity (Wagstaff & Bryson, 1997). TIZ suffers from rapid and extensive hepatic first-pass metabolism after oral administration which results in a poor drug bioavailability (34–40%). Also, TIZ exhibits a short elimination half-life of 2.5 h (Pendekal & Tegginamat, 2012, El-Mahrouk et al., 2014). The matters that necessitate frequent administration of TIZ which may result in poor patient compliance.

Transfersomes, the ultraflexible liposomes, composed of a bilayer former as phospholipid and edge activator. Transfersomes are more elastic than the conventional liposomes (Jain et al., 2003). Elasticity in these vesicles is attributed to the presence of an edge activator which is able to weaken the lipid bilayer of the vesicles and increase their deformability and flexibility. Edge activators are single chain surfactants with a high radius of curvature (Baldrick, 2000). Spans, Tweens, dipotassium glycyrrhizinate, sodium cholate and sodium deoxycholate were employed as edge activators (Cevc & Blume, 2003). Transfersomes, being deformable, can squeeze themselves through pores in the biological membranes, which are too much smaller than their own diameters.

Rectal route offers a useful, noninvasive alternative route of administration when local or systemic effect is intended. The rectum provides a relatively constant environment for drug delivery that allows a constant steady-state concentration of drug in plasma and partially avoids gastrointestinal (GI) absorption difficulties and hepatic first-pass metabolism (El-Leithy et al., 2010).

However, conventional solid suppositories are usually accompanied with discomfort that may lead to poor compliance and patient refusal. Moreover, if they lack mucoadhesion, conventional suppositories might reach the end of the colon. Therefore, incorporated drugs may be exposed to first-pass metabolism (Huang et al., 1987; Özgüney et al., 2014). Therefore, another rectal dosage form that is easy to administer and with mucoadhesive properties has to be used. So mucoadhesive *in situ* gels were used. The potential of thermosensitive *in situ* gels as a drug delivery system has been widely studied. They were investigated for use as delivery system for rectal, topical, nasal, ophthalmic, subcutaneous and intraperitoneal administration (Miyazaki et al., 1998).

The aim of the present study was to assess the possibility of the development of a new vesicle-loaded gelling system as a carrier for the rectal delivery of TIZ as an effective treatment for spasticity, overcoming the drawbacks of oral delivery. This was achieved by comprehensive *in vitro* and *in vivo* evaluation of the developed systems in an attempt to attain an optimized, effective and promising delivery system.

## Material and methods

### Materials

TIZ was obtained as a gift sample from Sigma Pharmaceutical Industries (Egypt). L-α-phosphatidylcholine (PC), cholesterol, sorbitan monooleate (Span 80), sodium deoxycholate (SDC), polyoxyethylene lauryl ether (Brij 35), poloxamer 407 (P407), poloxamer 188 (P188), hydroxypropyl methylcellulose (HPMC, K15M) were purchased from Sigma-Aldrich (St Louis, MO). Triton X-100 and polyoxyethylene sorbitan monooleate (Tween 80) were purchased from Loba Chemie (India). Dialysis bags with a molecular weight cutoff of 12 000 Da were purchased from SERVA Electrophoresis GmbH (Heidelberg, Germany). Tolterodine was kindly supplied from Sabaa Pharma (Egypt). All other ingredients used were of analytical grade.

### Preparation of TIZ-loaded transfersomal vesicles

Different transfersomal formulations were prepared by conventional rotary evaporation method described by Cevc et al. (1997) with some modification. Briefly, the lipid phase consisted of phospholipid, edge activators and cholesterol was dissolved in chloroform (5 ml). The organic solvent was removed under vacuum using a rotary evaporator (Stuart, RE300, Wolf Laboratories, UK) in a 55 °C water bath at 100 rpm. The formed film was maintained for 2 h in a desiccator under vacuum for complete removal of traces of solvent. The film was then hydrated with 10 ml phosphate buffer solution containing 10 mg TIZ and allowed to rotate at 80 rpm for 1 h under normal pressure. For particle size reduction, the obtained transfersomal suspensions were then sonicated for 30 min using a bath sonicator (Sonix IV ss-series, North Charleston, SC). Formulations were then kept in refrigerator for further studies (Al-Mahallawi et al., 2014; González-Rodríguez et al., 2016).

Table 1 demonstrates the composition of the prepared TIZ-loaded transfersomes, where Span 80, Tween 80, Brij 35 and SDC were used as edge activators along with L-α-PC as a bilayer former.

**Table 1.** Composition, entrapment efficiency, vesicle diameter and *ex vivo* permeation parameters of TIZ-loaded nanotransfersomes.

Formula	EA used	Composition (PC:EA)	EE (%)	Vesicle diameter (nm)	Q_24h_ (μg/cm)	J (μg/cm^2^ h)	Lag time (min)	Kp (cm/h)
F1	Span 80	95:5	60.16 ± 1.59	253.97 ± 6.6	198.36 ± 3.55	8.27 ± 0.15	30 ± 3.14	0.004469 ± 0.0007
F2	Span 80	85:15	56.74 ± 0.97	237.66 ± 7.1	222.80 ± 4.19	9.28 ± 0.18	23 ± 1.08	0.004873 ± 0.0009
F3	Span 80	75:25	58.83 ± 1.74	191.90 ± 3.5	166.19 ± 2.89	6.92 ± 0.13	24 ± 1.43	0.003899 ± 0.0008
F4	Tween 80	95:5	55.63 ± 2.04	198.87 ± 2.3	280.71 ± 2.20	11.69 ± 0.09	28 ± 2.13	0.01624 ± 0.0039
F5	Tween 80	85:15	52.39 ± 1.13	150.33 ± 2.8	411.49 ± 5.33	17.15 ± 0.22	11 ± 1.99	0.03628 ± 0.0078
F6	Tween 80	75:25	45.80 ± 1.55	145.03 ± 3.6	329.69 ± 4.71	13.74 ± 0.19	13 ± 3.60	0.01764 ± 0.0112
F7	Brij 35	95:5	43.21 ± 2.33	175.47 ± 1.1	324.67 ± 4.58	13.53 ± 0.19	15 ± 2.78	0.02668 ± 0.0130
F8	Brij 35	85:15	42.83 ± 1.45	149.73 ± 1.7	336.85 ± 3.48	14.04 ± 0.15	9 ± 1.72	0.03552 ± 0.0085
F9	Brij 35	75:25	39.18 ± 1.99	148.27 ± 2.2	333.79 ± 5.11	13.91 ± 0.21	10 ± 2.22	0.03073 ± 0.0127
F10	SDC	95:5	64.59 ± 1.40	253.23 ± 5.1	222.62 ± 3.25	9.28 ± 0.14	32 ± 3.01	0.01183 ± 0.0083
F11	SDC	85:15	44.33 ± 1.17	144.10 ± 8.6	233.96 ± 4.74	9.75 ± 0.20	19 ± 2.95	0.02419 ± 0.0074
F12	SDC	75:25	42.49 ± 2.18	255.55 ± 2.1	210.25 ± 3.69	8.76 ± 0.15	23 ± 1.44	0.01309 ± 0.0059
TIZ solution	–	–	–	–	105.54 ± 4.13	4.39 ± 0.18	13 ± 2.56	0.00187 ± 0.0004

PC: phosphatidylcholine; EA: edge activator; SDC: sodium deoxycholate.

Q_24h_: Cumulative amount of TIZ permeated per unit area; J: The flux of the drug; Kp: permeation coefficient.

Listed data are mean values ± SD (*n* = 3).

### Characterization of TIZ vesicles

#### Determination of TIZ entrapment efficiency (EE %)

First, free TIZ was separated by cooling centrifugation at 22 000 rpm for 1 h using cooling centrifuge (Sigma, 3–30K, Germany) at 4 °C. The obtained precipitate was washed twice with distilled water and re-centrifuged again to ensure complete removal of the free un-entrapped drug. The amount of entrapped TIZ was determined by disruption of the separated vesicles with 1% w/v Triton X-100 in phosphate buffer (pH 7.4) with sonication and heating to about 60 °C to ensure complete lysis of the vesicles. Furthermore, it was centrifuged for 20 min at 8000 rpm for precipitation of any debris. After a suitable dilution with phosphate buffer solution (pH 7.4), the solution was analyzed spectrophotometrically for the drug content at 319 nm using spectrophotometer (Jasco V-530, Japan). TIZ EE% was calculated using the following formula (Ghanbarzadeh et al., 2015):

$$\mathrm{EE}\%\;=\;\frac{\mathrm{Entrapped}\;\mathrm{drug}}{\mathrm{Total}\;\mathrm{drug}}\;\times \;100$$

#### Determination of particle size and polydispersity index (PDI)

The mean diameter and PDI of the prepared vesicles were estimated using the dynamic light scattering method (Mahmood et al., 2014). One milliliter transfersomal suspension was diluted to 10 ml with distilled water and measured by a Malvern Zetasizer at 25 °C (Zetasizer Nano ZS, Malvern Instruments, Malvern, UK). Three replicates of each sample were taken.

### *In vitro* release studies

The *in vitro* release profiles of TIZ-loaded transfersomal formulations were conducted and compared with the free drug (FG) solution using a fabricated vertical diffusion Franz cells with an effective diffusion area of 5 cm^2^. An accurate volumes of the transfersomal suspensions and drug solution, equivalent to 3 mg of drug, were placed in donor compartment and allowed to diffuse through a presoaked dialysis membrane (Mol. Wt cutoff = 12 000 Da) into 50 ml phosphate buffer solution with pH 7.4 (El-Kamel & El-Khatib, 2006) as a receptor medium. The receptor compartment was maintained at 37 ± 0.5 °C and stirred by a magnetic bar at 100 rpm.

At predetermined time intervals along 8 h (480 min) test period, 1 ml of samples was withdrawn from the receptor medium, replaced by an equal volume of buffer, filtered and analyzed spectrophotometrically at 319 nm. All experiments were done in triplicate and mean values ± SD were determined (El-Mahrouk et al., 2014; Abdelrahman et al., 2015).

The obtained release data were analyzed kinetically by different models; zero-order model, first-order model and Higuchi diffusion model. The correlation coefficient values (*R*^2^) were determined to find the model best fit the mechanism of TIZ release (Yang et al., 2015).

### *Ex vivo* permeation studies

Prepared transfersomal formulations were subjected to *in vitro* permeation study and permeation profile of TIZ was established. The study was performed according to the procedures previously described by Kamel et al. (2013) with slight modifications using a fabricated Franz diffusion cell stirred at 100 rpm for up to 24 h with cattle rectum as a diffusion membrane and temperature maintained at 37 ± 0.5 °C and the receptor compartment contains 50 ml phosphate buffer solution pH 7.4 (El-Kamel & El-Khatib, 2006).

At a predetermined time interval, 1 ml sample was withdrawn and replaced with equal volume of freshly prepared phosphate buffer to maintain a constant volume. Samples were filtered, suitably diluted and spectrophotometrically analyzed at 319 nm for the cumulative amount permeated of TIZ. The permeation of TIZ from aqueous drug solution was investigated in the same way. All experiments were done in triplicate and the mean values ± SD are listed in Table 1.

The cumulative amount of TIZ permeated was plotted as a function of time and rectal permeation parameters such as cumulative amount of drug permeated per rectum unit area during 24 h (Q_24_ in μg/cm^−2^), flux (J in μg/cm^2^/h, Equation 1), permeability coefficient (Kp in cm/h, Equation 2) were calculated. Lag time can be determined from the X-intercept of the linear portion of the plot.

(1) $$J\;=\;\frac{\mathrm{Amount}\;\mathrm{of}\;\mathrm{drug}\;\mathrm{permeated}}{\mathrm{Time}\;\times \;\mathrm{area}\;\mathrm{of}\;\mathrm{the}\;\mathrm{membrane}}$$

(2) $$\mathrm{Kp}\;=\;\frac{\mathrm{dQ}/\mathrm{dt}}{AC_{d}}$$

where *dQ*/*dt* = amount of drug/time which obtained from the slope of the straight portion of the plot, *A* is the diffusion area (5 cm^2^) and *C_d_* is the concentration of the drug in the donor compartment (Al-Mahallawi et al., 2014; González-Rodríguez et al., 2016).

### Selection of the optimized formula

The optimized formula of transfersomal suspension was selected on the basis of a good EE%, optimal vesicle size, high percentage of drug released and maximum cumulative amount permeated. The selected formula was characterized by transmission electron microscopy (TEM) and eventually incorporated into thermosensitive gel.

### Transmission electron microscopy

Visualization and morphological examination of the selected transfersomal formula F5 was conducted by means of TEM. One drop of transfersomal dispersion was dropped on a carbon grid as a thin film and allowed to dry. The film was then negatively stained with 1% phosphotungstic acid; excess liquid was removed by filter paper and finally allowed to dry. The air-dried sample was examined by TEM analyzer operating at an accelerating voltage of 80 kV (Joel, Tokyo, Japan) (Abdelrahman et al., 2015; Aboud et al., 2015).

### Preparation of thermosensitive *in situ* gel

The selected transfersomal formula F5 was incorporated in a thermosensitive *in situ* gel with the composition of (21:3:0.8%) for P407:P188:HPMC. *In situ* gel formulations were prepared on a weight/volume basis using the cold method described by Schmolka et al. (Schmolka, 1972; Pan & Yang, 2011) with slight modification.

Briefly, mucoadhesive polymer was weighed, slowly sprinkled on accurately measured volume of freshly prepared transfersomal suspension of F5 for preparation of transfersomes-loaded *in situ* gel (TG).

Mixture was gently stirred using magnetic stirrer till completely dissolve. Then, mixture of P407 and P188 (21:3%w/v) was dispersed, gently mixed and stored in refrigerator at 4 °C till poloxamer granules were completely dissolved and a clear homogenous solution was obtained.

A similar gel formulation of free drug (FG) was prepared using the same procedure. The concentration of the drug was maintained as 0.2% in both FG and TG formulations.

### Histopathological evaluation

This study aimed to investigate the effect of different *in situ* gelling formulations FG and TG on the integrity of the rectal mucosa. The study was performed according to the guidelines of the local animal ethical committee of Beni-Suef University. Nine Albino Wister rats weighing 150–200 g were divided into three groups (three rats per each group) and fasted for 24 h prior to the experiment with free access to water to reduce the fecal matter in the rectum.

Group A was the control, whereas groups B and C received FG and TG, respectively. At 8 h after administration; animals were sacrificed, rectum was isolated, rinsed with saline and fixed in 10% formalin buffer. Furthermore, rectum segments were embedded in paraffin and cut into slices. The slices were stained with hematoxylin and eosin and observed under light microscope (El-Leithy et al., 2010; Ud Din et al., 2015). This evaluation was performed in a blinded manner by an experienced veterinary histopathologist.

### Pharmacokinetic study

#### Animals

For each examined formulation, three New Zealand white male rabbits, weighing 2–2.5 kg were used. Rabbits were fasted for 24 h prior to the experiment with free access to water to avoid defecation during the experiment. Rabbits were conscious during dosing and the whole duration of the experiment. Rabbits were held in restrainers during blood sampling.

This study was performed according to the guidelines of the local animal ethical committee of Beni-Suef University.

#### Study design

A single dose equivalent to 1 mg/kg TIZ was administered to rabbits using randomized crossover design in three stages with 1 week washout period in between.

#### Dosage and dose administration

The three groups of rabbits received a dose of 1 mg/kg of TIZ from different formulations irrespective of the route of administration. Formulations used were as follows: TIZ solution in purified water for oral administration, *in situ* gel of FG and *in situ* gel of transfersomal formula F5 (TG) both for rectal administration. Gel formulations were administered 4 cm above the anus using a stomach Sonde needle fitted to a disposable plastic syringe. All formulations contained drug as 2 mg/ml.

#### Sample collection

At several intermissions (0, 0.5, 1, 2, 4, 8, 12 and 24 h), 2 ml blood samples was collected into heparinized tubes from the marginal ear vein of the rabbits. Blood samples were centrifuged at 5000 rpm for 30 min for complete separation of the plasma which were stored at −20 °C till analysis.

#### Samples preparation for analysis

A calibration curve of TIZ in plasma was conducted over the range of 0.1–200 ng/ml. A solvent extraction procedure for extraction of drug was performed. Fifty microliters of tolterodine (as internal standard) were mixed with 0.5 ml plasma and vortexed for 30 sec. In all samples 5 ml of ethyl acetate:hexane (90:10 v/v) mixture was added and samples were vortexed again for 1 min and centrifuged for 5 min at 4000 rpm. Furthermore, the organic liquid layer was transferred to another tube and evaporated at 45 °C till dryness. Dry residues were reconstituted with 150 μl mobile phase, methanol:0.1% formic acid 80:20 (v/v). Finally, 5 μl was injected on the column for analysis using the autosampler.

#### LC-MS/MS assay of TIZ

Plasma samples were analyzed for TIZ using a reproducible, sensitive and accurate liquid chromatography-tandem mass spectroscopy (LC-MS/MS) method. Method was developed and validated prior to the analysis. LC system (agilent, Germany) with a quaternary pump and autosampler was coupled with a triple quadripole MS/MS detector. The chromatographic separation was performed on akinetex C18 column 50 × 4.6 mm and particle size of 2.6 μ (Phenomenex, Inc., Torrance, CA). The mobile phase consisted of methanol:0.1% formic acid 80:20 (v/v) was delivered into the MS’s electrospray chamber at a flow rate of 0.4 ml/min. The ion spray voltage was adjusted at 3000 V. The common parameters were as follows: nebulizer gas pressure was 45 psi, gas temperature was 275 °C and gas flow rate was 8 l/min.

The analysis was carried out at the multiple reaction monitoring (MRM) mode, and its MS parameters were as follows: precursor ion (Da) was 254.1 and 326.5 for TIZ and tolterodine, respectively, whereas product ion (Da) was 44.2 and 147.2 for TIZ and tolterodine, respectively (El-Mahrouk et al., 2014).

### Pharmacokinetic analysis

Pharmacokinetic parameters were estimated from plasma data using computer program WinNonlin (version 1.5, Scientific consulting, Inc., Rockville, MD). Non-compartmental pharmacokinetic model was adopted for the calculation of the maximum drug concentration (*C*_max_, ng/ml) and time needed to reach this concentration (*T*_max_, h) from each rabbit plasma concentration–time curve. Trapezoidal rule method was adopted for calculation of the area under the curve (AUC) from 0 to 24 (AUC_0–24_, ng h/ml) and from 0 to infinity (AUC_0–∞_, ng h/ml). The latter can be calculated from AUC_0–24_, where AUC_0–∞_ = AUC_0–24_ + C*/k; C* is the last measured concentration and k is the terminal elimination rate constant.

Terminal elimination half-life (*t*_1/2_) can be calculated from k as *t*_1/2 _=_ _0.693/k. Finally, relative bioavailability (F_rel_) for both rectal formulations can be assessed, considering oral formulation as a standard, from the following formula:

$$F_{\mathrm{rel}}\;=\;\frac{\mathrm{AUC}\;\mathrm{test}}{\mathrm{AUC}\;\mathrm{standard}}\;\times \;100$$

### Statistical analysis

Statistical analysis of the results was performed using one-way analysis of variance (ANOVA) followed by the Tukey multiple comparisons test. Mean differences were considered statistically significant at a level *p* < 0.05. All calculations were performed using the computer program SPSS 16 (SPSS, Chicago, IL). All experiments were done at *n* = 3 and data were presented as mean value ± SD.

## Results and discussion

### Entrapment efficiency

Being hydrophilic, TIZ would be incorporated into the internal aqueous core of vesicles as suggested by Lopes et al. (2004). The EE% of TIZ in different transfersomal formulations was in the range of 39.18–64.59%, as shown in Table 1. EE% varied with different types of EAs used and its concentration relative to PC. With regard to PC:EA ratio, the ratio of 95:5% w/w gave the highest EE% irrespective to the type of EAs. By increasing the concentration of EAs to 15%, the EE% was decreased and further increase in the concentration of the EAs was accompanied with a further decrease in the EE%.

This could be attributed to the growth in the vesicles size as a result of association of EA molecules with phospholipid bilayer. This growth was, initially, accompanied with incorporation of a high amount of drug. Further addition of EA may lead to formation of pores in the lipid bilayer. Further increase beyond 15% lead to the formation of rigid small-sized micelles along with vesicles with the consequence of a lower EE (El-Zaafarany et al., 2010).

Furthermore, the ratio of maximum EE (95:5%) was compared in formulations with different EAs for investigation of the effect of different types of EAs on EE. SDC showed the highest EE (64.59%) followed by Span 80 (60.16%), Tween 80 (55.63%) and finally Brij 35 (43.21%). Generally, these results could be explained on the basis of hydrophile-lipophile balance (HLB) values of the EAs used. They are 16.7, 4.3, 15 and 16.9 for SDC, Span 80, Tween 80 and Brij 35, respectively. HLB values depend on the alkyl chain length of EAs, where the longer the alkyl chain, the lower HLB values and the higher EE% (Hao et al., 2002; Abdelbary & El-Gendy, 2008). The matter that is consistent with our results. Unexpectedly, SDC-based transfersomes gave the highest EE.

A possible explanation may be due to the anionic nature of deoxycholate that impart negative charge on the vesicles leading to generation of a high repulsive force between the lamellae. Thus, the size of the internal aqueous core increased with the consequence of higher EE of the hydrophilic drug TIZ (González-Rodríguez et al., 2016).

Moreover, cholesterol was added with a fixed amount to all formulations with the intent of improving the encapsulation efficiency of hydrophilic drugs by stabilizing the bilayer membrane and so prevent leakiness of the drug from the aqueous core (Abdelbary & El-Gendy, 2008; Elhissi et al., 2012; González-Rodríguez et al., 2016).

### Particle size and PDI

The mean size of the prepared transfersomal formulations was measured and found to be in the range of 144.10–282.20 nm as shown in Table 1. The results indicated that particle size decreases on increasing the concentration of EAs. Thus, higher concentration of EAs provided particles with a smaller size that might be attributed to the availability of a high amount of EA that can cover the particle surface, decreasing their interfacial tension and so decreasing their particle size. Similar results were obtained by Al-Mahallawi et al. (2014) and Salama et al. (2012).

On the other hand, particle size was greatly affected by the type of EA used. Upon comparing the ratio (95:5%), particle size was 253.79, 198.87, 175.47 and 253.23 nm for Span 80, Tween 80, Brij 35 and SDC, respectively. One way to interpret these results is to consider their HLB values.

They are 4.3, 15, 16.9 and 16.7 for Span 80, Tween 80, Brij 35 and SDC, respectively. Particle size increases along with decrease in the HLB value. As the EA with higher HLB undergoes interaction with the inner aqueous phase which decrease the particle size. On the other hand, EA with low HLB value undergoes interaction with the lipid head groups of the membrane increasing the packing density of the bilayer and increasing the surface free energy.

As a result, fusion between the lipid bilayer would occur and so the particle size is increased. Thus, the longer the chain length of EA, the lower the HLB value and the larger the particle size. SDC-based vesicles seem to deviate from this explanation that could be attributed to the anionic nature that imparts negative charge on the vesicles leading to generation of a high repulsive force between the lamellae. Thus, the size of the internal aqueous core increased with the consequence of higher particle size, as previously mentioned.

Our results agreed with previous studies demonstrated the effect of the HLB of surfactant on the particle size of drug-loaded transfersomes (Abdelbary & El-Gendy, 2008; Khan et al., 2014; Ali et al., 2015), which all encapsulate water-soluble drugs. But disagree with other studies incorporating water insoluble drugs (Yusuf et al., 2014; Aboud et al., 2015).

PDI was used to evaluate the homogeneity of the vesicular dispersions. A value of 0 specifies a monodispersed particles within the system, whereas a value of 1 specifies a highly polydispersed system (Zeisig et al., 1996). PDI of TIZ-loaded transfersomes ranged from 0.252 to 0.638 that indicates a good size distribution and satisfying homogeneity of the formulations (data not mentioned).

### *In vitro* release studies

**Figure 1.**
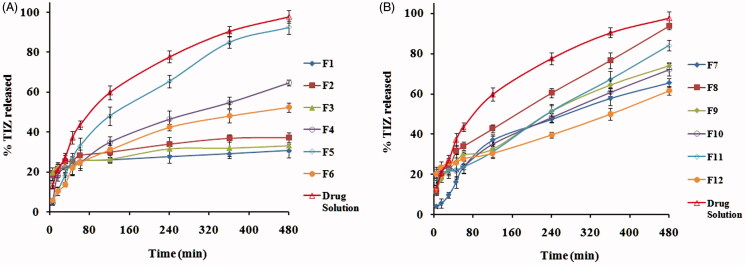
*In vitro* release profiles of TIZ from drug solution and transfersomal formulae: (A) F1–F6 and (B) F7–F12.

The percentage of TIZ released after 8 h (Q_8h_) from different transfersomal formulations, in comparison with that of TIZ solution as a control, is represented graphically in Figure 1(a and b). The release of TIZ from control is markedly faster than that from vesicles which may be attributed to the well-known reservoir effect of vesicular systems that provides a sustained release profile (Ghanbarzadeh & Arami, 2013; Shaji & Lal, 2014). Moreover, the high Q_8h_ of control (97.7%) as well Q_8h_ of some formulations clearly confirms that the membrane used in the experiment did not hinder the drug release and the sink condition was completely accomplished.

Generally, the release profile of TIZ seems to be a biphasic processes and the Q_8h_ seems to increase with increasing the EA concentration from 5 to 15% and decrease with a further increase in EA concentration to 25%, a common phenomenon observed with all EAs.

Biphasic release might be attributed to the initial rapid release of the surface-adsorbed free TIZ followed by sustained release of the drug from the vesicle core (El-Zaafarany et al., 2010). The change of Q_8h_ with EAs concentration might be attributed to the hindrance of drug release at both low (5%) and high concentration (25%) of EAs caused by the presence of the membrane bilayer in a more ordered, less leaky form which hinder drug release at low concentration and loss of the vesicular structure along with formation of a rigid mixed micelles at high concentration. Mixed micelles were reported to be less sensitive to the water activity gradient than transfersomes (El-Maghraby et al., 2000; Jain et al., 2003).

On the other hand, the PC:EA with the ratio (85:15%. w/w) produces vesicles with a higher Q_8h_ due to association of EAs molecules with the phospholipid bilayer to provide better partitioning of the drug (Gupta et al., 2012). Thus, formulations, prepared using this ratio, were selected to demonstrate the effect of different EAs on Q_8h_.

The Q_8h_ was 93.69, 92.52, 84.03 and 37.32%, respectively, for Brij 35, Tween 80, SDC and Span 80 based transfersomes. One way to explain these results is to consider the fact that vesicles exhibit alky chain-length-dependent drug release. The higher the EA chain length, the lower the drugs release. The difference in the alkyl chain might induce variations in the molecular ordering of the vesicles that affect the release rate (Hosny & Hassan, 2014). Another way to explain these results is to consider their particle size, where small particle size provides a larger surface area exposed to the release medium and thus enhance the drug release (Abdelbary & El-Gendy, 2008; El-Zaafarany et al., 2010; Aboud et al., 2015).

Our results agree with El-Zaafarany et al. who reported that the release of diclofenac sodium from transfersomes was in the order: Tween 80 > SDC > SC > Span 80 > Span 85 (El-Zaafarany et al., 2010) and agree with Abdelbary and El-gendy who reported that the release of gentamicin sulfate from niosomes was in the order: Brij 35 > Tween 80 > Tween 60 (Abdelbary & El-Gendy, 2008).

But, disagree with studies reported by Jain et al. on dexamethasone-loaded transfersomes where the release rate was in the order: Span 80 > SDC > Tween 80 (Jain et al., 2003). These observations may be attributed to the difference in the nature of different drugs used.

Linear regression analysis of the obtained release data revealed that TIZ was released from majority of vesicles by diffusion controlled mechanism except F10, F12 and drug solution which followed first-order kinetics.

### *Ex vivo* permeation studies

Permeation of molecules across biological membranes is a multistep, multifactorial phenomenon depending on various types of chemical, physical and biological interactions. However, the *ex vivo* permeation studies provide a valuable insight on the *in vivo* performance of many products (Ammar et al., 2009).The rectum is known to have a similar epithelium to that of the upper GI tract and the predominant mechanism of permeation through the rectal mucosa appears to involve transcellular passage across the cell membrane (Kamel et al., 2013). The calculated permeation parameters, listed in Table 1, were used to assess the permeation efficiency of the tested transfersomal formulations against TIZ solution.

Generally, transfersomal formulations showed a higher permeation, the matter that could be attributed to their composition of phospholipid and EAs. EA imparts deformability to the membrane bilayer of the vesicles, thereby facilitating their passage across rectal epithelial. Moreover, phospholipids were reported to have high affinity for biological membranes that contribute to enhancement of the permeation efficiency (El-Zaafarany et al., 2010; González-Rodríguez et al., 2016).

Based on Q_24h_, transfersomes with high (25% w/w) and low (5% w/w) concentration of EAs were not beneficial in vesicular drug delivery, whereas those containing the middle (15% w/w) concentration of EAs showed a higher permeability profiles, irrespective of the EA type. As previously mentioned, at low concentration of EA transfersomes showed a large size, which negatively influence the permeation process. At high concentration of EA, small rigid micelles were formed, which were reported to be less deformable and less effective in drug delivery than transfersomes (Jain et al., 2003; Aboud et al., 2015).

Upon comparing the ratio of maximum Q_24h_ (85:15), formulation containing Tween 80 showed the highest Q_24h_, followed by Brij 35, SDC and finally Span 80 containing transfersomes. A possible explanation of these results is the difference in the alkyl chain length of the used EA, where the lower the chain length of the EA in the formulations, the higher the permeation of the drug (Kamel et al., 2013).

Moreover, difference in the particle size between the mentioned formulations might play a role in the permeation across the biological membrane. The reduced dimensions of the vesicles could positively influence their permeation (Bragagni et al., 2012). These results correlate well with those obtained from the *in vitro* release studies.

### Morphology of TIZ vesicles

**Figure 2.**
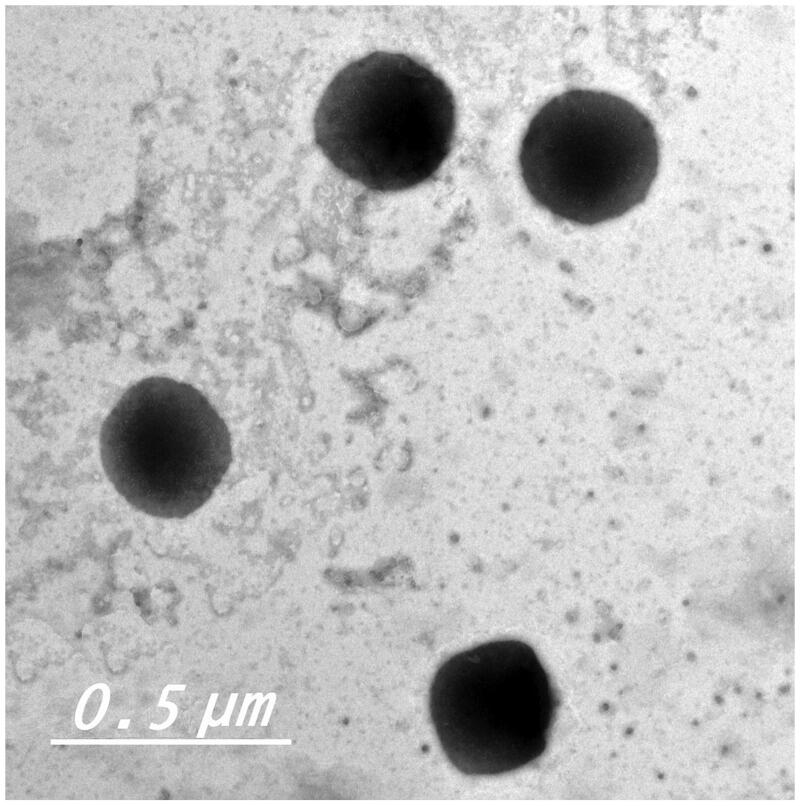
Transmission electron micrograph of freshly prepared transfersomal formula F5.

TEM analysis provides a useful mean for examination of the morphology of colloidal systems and also in confirming the results obtained by size analysis (Lim et al., 2014). Transmission electron micrograph of the optimum formula displayed a non-aggregated, well-identified unilamellar and spherical-shaped vesicles with a large internal aqueous core (Figure 2).

### Histopathological studies

**Figure 3.**
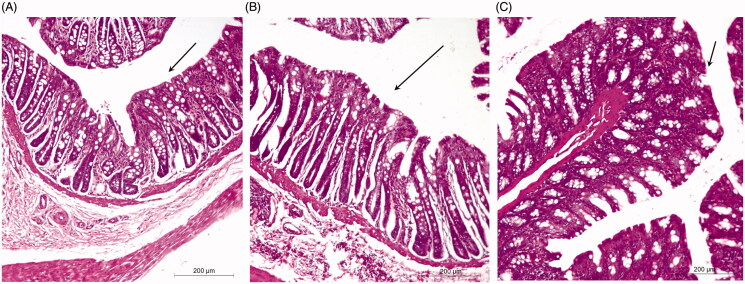
Morphology of rabbit rectal mucosa: (A) control, (B) 8 h after administration of FG and (C) 8 h after administration of TG.

The histopathological evaluation was performed to inspect any irritation or damage to the rectal mucosa of rats after administration of TG and FG as compared to the control (Figure 3). In this study, normal healthy rectal mucosa without administration was used as a control (Figure 3a). Rectal tissues of group B showed focal massive leukocytosis inside the submucosa associated with hemorrhage and mild congested blood capillaries that may be attributed to the acidic nature of the free TIZ (Figure 3b).

On the other hand, rectal tissues of group C showed no signs of damage or irritation and tissues appeared to be more or less normal as compared to the control (Figure 3c). Thus, current evaluation suggests that transfersomes-loaded *in situ* gel might therefore be regarded as safe.

### *In vivo* pharmacokinetics studies

The LC–MS/MS method was used to estimate the pharmacokinetic parameters of TIZ in rabbit plasma to investigate the *in vivo* behavior of the TG formula as compared with FG formula and oral drug solution as a standard. The LC–MS/MS method was validated and confirmed good linearity from 0.1 to 200 ng/ml.

**Figure 4.**
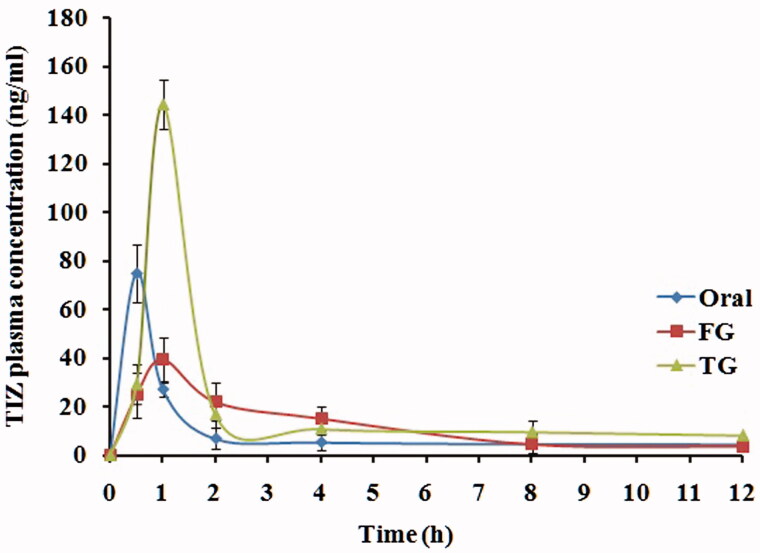
Mean TIZ concentrations in rabbit plasma following administration of oral solution, rectal TIZ *in situ* gel (FG) and rectal transfersomal *in situ* gel (TG).

The mean plasma concentration–time profiles of TIZ following administration of oral solution, rectal FG and rectal TG are illustrated in Figure 4 and the corresponding pharmacokinetic parameters are outlined in Table 2. As shown, TIZ was immediately absorbed and reached maximal concentration, *C*_max_, of 75.04 ng/ml at 0.5 h after oral administration of the drug solution. Then, concentration of TIZ was remarkably decreased in the following hours.

**Table 2.** Pharmacokinetic parameters of TIZ in rabbit plasma following administration of oral solution, FG and TG.

	Formulae		
Pharmacokinetic parameters	Oral solution	FG	TG
*C*_max_ (ng/ml)	75.04 ± 11.86	43.16 ± 9.36	144.31 ± 11.12
*T*_max_ (h)	0.5 ± 0.00	0.75 ± 0.25	1 ± 0.00
K_e_ (h^−1^)	0.203 ± 0.07	0.0962 ± 0.001	0.0692 ± 0.0074
*t*_1/2_ (h)	3.41 ± 0.89	7.21 ± 0.04	10.13 ± 1.08
AUC_0–24_ (ng h/ml)	164.13 ± 7.58	204.23 ± 20.90	314.68 ± 12.72
AUC_0–∞_ (ng h/ml)	173.49 ± 16.61	239.83 ± 28.11	379.48 ± 21.92
MRT (h)	4.71 ± 0.99	10.93 ± 0.53	12.03 ± 2.03
F_rel_ (%)	100	138.24	218.73

*C*_max_: maximum drug concentration in plasma; *T*_max_: time to reach *C*_max_; K_e_: elimination rate constant; *t*_1/2_: terminal half-life; AUC_0–24_: area under plasma concentration–time curve from 0 to 24 h; AUC_0–∞_: total area under plasma concentration–time curve; MRT: mean residence time; F_rel_: relative bioavailability. Listed data are mean values ± SD (*n* = 3).

The maximum concentration of 43.16 ng/ml and 144.31 ng/ml was reached at 0.75 h and 1 h for FG and TG, respectively. Compared to oral solution, the relative bioavailability calculated from AUC_0–∞_ was found to be 218.73% and 138.24%, respectively, for TG and FG which is considered a remarkable enhancement in TIZ bioavailability.

This enhancement in bioavailability can be attributed to the rectal route of administration that bypass the extensive hepatic first-pass metabolism, whereas higher enhancement of TG, as compared to FG, can be explained by the permeation enhancing effect of transfersomal vesicles.

The significant increase in the half-life (*t*_1/2_) of both rectal formulations indicated retardation in the TIZ release caused by the use of gel system, whereas higher retardation exerted by TG formula may be attributed to the dual sustained effect caused by both vesicular and gelling system.

These results are in good agreement with the *in vitro* release data of the drug. In conclusion, the significant increase in AUC_0–∞_, F_rel_ and *t*_1/2_ of *in situ* gelling nanotransfersomal formulation of TIZ indicates that an excellent increase in the bioavailability and a sustained release of the drug were achieved.

## Conclusion

The optimum formula F5 with the composition 85:15% of PC:Tween 80 showed a relatively high EE%, small particle size and controlled release of TIZ over 8 h. The pharmacokinetic study revealed that the optimum formula increased the bioavailability of TIZ 2.18-fold when compared to oral drug solution. It also showed a sustained drug release with a *t*_1/2_ of 10 h. Thus, the developed transfersomal formula could be considered as a promising rectal delivery system of TIZ that could bypass the hepatic first-pass metabolism, enhancing TIZ bioavailability, control its release with the consequence of reduced frequency of TIZ administration and improved the patient compliance.
